# Clinical Impact and Prognostic Role of Triglyceride to High-Density Lipoprotein Cholesterol Ratio in Patients With Chronic Coronary Syndromes at Very High Risk: Insights From the START Study

**DOI:** 10.3389/fcvm.2022.874087

**Published:** 2022-04-13

**Authors:** Leonardo De Luca, Pier Luigi Temporelli, Furio Colivicchi, Lucio Gonzini, Maria Luisa Fasano, Massimo Pantaleoni, Gabriella Greco, Fabrizio Oliva, Domenico Gabrielli, Michele Massimo Gulizia

**Affiliations:** ^1^Department of Cardiosciences, Division of Cardiology, S. Camillo-Forlanini, Roma, Italy; ^2^Division of Cardiology, Istituti Clinici Scientifici Maugeri, Istituto di Ricovero e Cura a Carattere Scientifico (IRCCS), Gattico-Veruno, Italy; ^3^Division of Cardiology, S. Filippo Neri Hospital, Roma, Italy; ^4^ANMCO Research Center, Firenze, Italy; ^5^Division of Cardiology, Cardiac Rehabilitation Unit, S. Carlo Hospital, Potenza, Italy; ^6^Division of Cardiology, Santa Maria Nuova Hospital, Reggio Emilia, Italy; ^7^Division of Cardiology, Santo Spirito Hospital, Roma, Italy; ^8^Cardiovascular Department, Division of Cardiology, “A. De Gasperis”, ASST Grande Ospedale Metropolitano Niguarda, Milano, Italy; ^9^Division of Cardiology, Garibaldi-Nesima Hospital, Catania, Italy

**Keywords:** hypercholesterolemia, LDL-C, management, treatment, statin, chronic coronary syndrome

## Abstract

**Background:**

Several studies have reported that the combination of high TG and low HDL-C, as simplified by the TG/HDL-C ratio, was a predictor of cardiovascular disease independent of LDL-C level. Nevertheless, poor data are available on the predictive role of TG/HDL-C ratio in very high risk (VHR) patients with chronic coronary syndromes (CCS).

**Methods:**

Using the data from the STable Coronary Artery Diseases RegisTry (START) study, an Italian nationwide registry, we assessed the association between the TG/HDL-C ratio and baseline clinical characteristics, pharmacological treatment, and major adverse cardio-cerebrovascular events (MACCE) at 1 year in a large cohort of CCS patients at VHR.

**Results:**

VHR patients with both TG and HDL-C levels available were grouped in tertiles of TG/HDL-C ratio: low (TG/HDL-C ratio <2, *n* = 967), middle (TG/HDL-C ratio 2–3.3, *n* = 1,071) and high (TG/HDL-C ratio >3.3, *n* = 1,028). At 1 year from enrolment, 232 (7.6%) patients presented a MACCE, with a higher incidence in the higher tertile, even though not statistically significant (6.0, 8.2, and 8.4% in the low, middle and high tertile, respectively; *p* = 0.08). At multivariable analysis, the TG/HDL-C ratio in tertiles did not result an independent predictor of the MACCE (*p* = 0.29) at 1-year follow-up (HR: 1.30; 95% CI: 0.93–1.82; *p* = 0.12 middle vs. lower tertile, and HR: 1.22; 95% CI: 0.87–1.72; *p* = 0.25 higher vs. lower).

**Conclusions:**

In the present large, nationwide cohort of CCS patients at VHR a high TG/HD ratio did not emerge as independent predictor of MACCE at 1 year. Further studies with a longer follow-up are needed to better define the prognostic role of TG/HDL ratio in CCS.

## Introduction

Recent data suggested that concomitant high triglycerides (TG) and low high-density lipoprotein cholesterol (HDL-C) levels, as simplified by the TG/HDL-C ratio, characterize a specific cardio-metabolic profile known as atherogenic dyslipidemia, a predictor of coronary artery disease (CAD) development and elevated risk of cardiovascular adverse events, independent of low-density lipoprotein cholesterol (LDL-C) levels (1–4). The mechanisms by which a high TG/HDL-C ratio is associated with an increased cardiovascular risk seem to be related to metabolic syndrome, insulin resistance and consequent hyperinsulinemia, and to direct implications in endothelial damage and atherosclerosis (5, 6). The role of TG/HDL-C ratio for the long-term prediction of cardiovascular events has been reported in different clinical scenarios, such as diabetes mellitus (DM) (7), chronic kidney disease (CKD) (8), and acute coronary syndromes (ACS) (9).

Nevertheless, poor data are available on the predictive role of TG/HDL-C ratio in patients with established chronic coronary syndromes (CCS), particularly in those at very high risk (VHR). Therefore, using the data from the STable Coronary Artery Diseases RegisTry (START) study (10, 11), an Italian nationwide registry, we sought to assess the association between the TG/HDL-C ratio and baseline clinical characteristics, pharmacological treatment, and major adverse cardio-cerebrovascular events (MACCE) at 1 year in a large cohort of CCS patients at VHR.

## Methods

The design and main results of the START registry have been published previously (10). Briefly, the START was a prospective, observational, nationwide study aimed to evaluate the current presentation, management, treatment and quality of life of patients with CCS as seen by cardiologists in clinical practice in Italy, during a 3-month period (10). Enrolment was made at the end of outpatient or day-hospital visit or at hospital discharge. Data on baseline characteristics, including demographics, risk factors and medical history, were collected. Information on the use of diagnostic cardiac procedures, type and timing of revascularization therapy (if performed) and use of pharmacological or non-pharmacological therapies were recorded on an electronic case report form (CRF) at hospital discharge or the end of outpatient visit.

The Italian Association of Hospital Cardiologists (ANMCO) invited to participate all Italian cardiology wards, including university teaching hospitals, general and regional hospitals, and private clinics receiving patients with CCS. No specific protocols or recommendations for evaluation, management, and/or treatment have been put forth during this observational study (10).

All patients were informed of the nature and aims of the study and asked to sign an informed consent for the anonymous management of their individual data. Local Institutional Review Boards (IRB) approved the study protocol according to the current Italian rules.

One-hundred eighty-three cardiology centers included consecutive patients in the survey in different periods of 3 months between March 2016 and February 2017 (10).

All patients included into the analysis were evaluated for being at VHR according to the ESC/EAS clinical guidelines for the management of dyslipidemias [e.g., documented atherosclerotic cardiovascular disease including previous ACS, coronary revascularization, stable angina, stroke or transient ischemic attack, peripheral artery disease (PAD), DM with target organ damage or type 1 DM of long duration, severe CKD, a SCORE ≥10% for 10-year risk of fatal cardiovascular disease or familial hypercholesterolemia with atherosclerotic cardiovascular disease or another major risk factor] (12, 13).

Optimal medical therapy (OMT) has been defined as patients being prescribed aspirin or thienopyridine, β -blocker and a statin, at the maximum tolerated dosage (14). To be categorized as receiving OMT, individual patients must have been either prescribed or had reported contraindications to all medications in each category. Data on the use of angiotensin-converting enzyme inhibitors (ACE-I) or angiotensin II receptor blockers (ARB) were recorded and could be used to calculate their use among those patients in whom they were clinically indicated. Therefore, given that the guidelines for the management of CCS (14) recommend an ACE-I or ARB for some subgroups of patients, we also examined OMT including ACE-Is or ARBs, if indicated by an ejection fraction ≤40%, hypertension, DM or CKD (eligible patients).

In the present analysis, we considered the VHR patients with both TG and HDL- C available at enrollment.

### Clinical Events and Follow-Up

Patients were followed up by visits or telephone interviews by investigators at 1 year from enrolment. Interviews included questions related to the occurrence of events, planned and unplanned hospitalizations and persistence to pharmacological therapies prescribed at enrolment.

The primary outcome of the present analysis was the time to the first occurrence of MACCE, a composite of all-cause mortality, hospitalization for MI, HF, stroke/TIA or myocardial revascularization at 1-year follow-up.

### Statistical Analysis

The study cohort was stratified according the tertiles of TG/HDL-C ratio (<2; 2–3.3; >3). Categorical variables are presented as number and percentages and compared by the Chi-square test. Continuous variables are presented as mean and standard deviation (SD), except the TG levels, dosages of prescribed statins and time from revascularization to enrollment, which are reported as median and inter-quartile range (IQR) and were compared by the *t*-test or analysis of variance, if normally distributed, or by Mann-Withney *U*-test test or Kruskall-Wallis, if not.

A multivariable analysis (Cox regression) was performed in order to identify the independent predictors of MACCE at 1-year follow-up. We inserted in the model the TG/HDL-C ratio tertiles (low tertile as reference), and the following variables considered of clinical interest as covariates: age (continuous), gender, prior ACS, prior revascularization, DM, hypertension, hypercholesterolemia, history of HF, PAD, history of major bleeding, CKD, the achievement of LDL-C target according to ESC/EAS guidelines (13), use of OMT at discharge. When more than two categories were present, dummy variables were introduced to define a reference group. Patients without a MACCE event was censored at their time of last observation. Kaplan–Meier curves were produced for the MACCE at 1- year follow-up and compared by log-rank test.

A *p* < 0.05 was considered statistically significant. All tests were 2-sided. Analyses were performed with SAS system software, version 9.4.

## Results

From the 5,070 consecutive patients with CCS enrolled in the registry, 4,751 (94.0%) were classified as VHR. Among this latter group of patients, 3,066 (64.5%) had both values of TG and HDL-C at baseline. Patients at VHR with both TG and HDL-C levels available were younger, less frequently females and more often active smokers, compared to VHR patients with TG and/or HDL-C not available, and that were also excluded from the analysis (Supplementary Table 1).

The TG/HDL ratios were normally distributed in the study population and the mean value was 3.10 ± 2.18 mg/dl. Patients at VHR with both TG and HDL-C levels available were grouped in tertiles of TG/HDL-C ratio: low (TG/HDL-C ratio <2, n = 967), middle (TG/HDL-C ratio 2–3.3, *n* = 1,071) and high (TG/HDL-C ratio >3.3, *n* = 1,028). Baseline characteristics of patients in the three groups are shown in Table 1. Although patients in the highest tertile, predominantly males, were significantly younger, they presented more often risk factors such as DM, smoking, hypertension, CKD and higher levels of glycaemia, serum uric acid, total and LDL cholesterol compared to patients in the other tertiles (Table 1). The median time between coronary revascularization and enrolment was 177 (IQR 35-1018) days.

**Table 1:** Baseline clinical characteristics of VHR patients with TG and HDL-C available.

	**Overall *n* = 3,066**	**Tertiles of TG/HDL-C ratio**			***P***
		**Low (<2) *n* = 967**	**Middle (2–3.3) *n* = 1071**	**High (>3.3) *n* = 1028**	
Age (years), mean ± SD	67 ± 10	68 ± 10	68 ± 10	66 ± 10	<0.0001
Females, n (%)	555 (18.1)	214 (22.1)	183 (17.1)	158 (15.4)	0.0003
BMI (kg/m^2^), mean ± SD	27.4 ± 4.0	26.3 ± 3.6	27.4 ± 3.9	28.4 ± 4.1	<0.0001
**Risk factors and comorbidities**, ***n*** (%)
Active smokers	572 (18.7)	143 (14.8)	190 (17.7)	239 (23.3)	<0.0001
Hypercholesterolaemia	2,370 (77.3)	725 (75.0)	806 (75.3)	839 (81.6)	0.0003
Diabetes mellitus	1,025 (33.4)	247 (25.5)	342 (31.9)	436 (42.4)	<0.0001
Hypertension	2,430 (79.3)	736 (76.1)	844 (78.8)	850 (82.7)	0.001
Chronic renal dysfunction^*^	365 (11.9)	87 (9.0)	114 (10.6)	164 (16.0)	<0.0001
Peripheral artery disease	309 (10.1)	96 (9.9)	103 (9.6)	110 (10.7)	0.70
COPD	359 (11.7)	115 (11.9)	113 (10.6)	131 (12.7)	0.29
Malignancy	186 (6.1)	67 (6.9)	59 (5.5)	60 (5.8)	0.38
**Cardiovascular history**, ***n*** **(%)**
Previous stroke/TIA	180 (5.9)	55 (5.7)	57 (5.3)	68 (6.6)	0.43
History of major bleeding	61 (2.0)	14 (1.5)	24 (2.2)	23 (2.2)	0.35
Atrial fibrillation	418 (13.6)	139 (14.4)	157 (14.7)	122 (11.9)	0.13
History of heart failure	415 (13.5)	117 (12.1)	138 (12.9)	160 (15.6)	0.06
Prior MI	2,190 (71.4)	698 (72.2)	774 (72.3)	718 (69.8)	0.39
Previous PCI/CABG	2,582 (84.2)	798 (82.5)	905 (84.5)	879 (85.5)	0.18
***Haemodynamic parameters, mean** **±SD; median (IQR)***
LVEF, % (*n* = 2,891)	50.0 ± 10.0	54.5 ± 9.8	54.0 ± 10.1	53.5 ± 9.9	0.10
SBP, mmHg	130 ± 16	130 ± 17	130 ± 16	130 ± 16	0.68
DBP, mmHg	76 ± 9	76 ± 9	76 ± 9	77 ± 9	0.03
HR, bpm	66 ± 11	65 ± 10	66 ± 12	67 ± 11	<0.0001
Hb, g/dL (*n* = 2,896)	13.6 ± 1.7	13.7 ± 1.6	13.6 ± 1.6	13.6 ± 1.8	0.87
Creatinine, mg/dL (*n* = 2,886)	1.1 ± 0.5	1.0 ± 0.4	1.0 ± 0.6	1.1 ± 0.6	<0,.0001
Total cholesterol, mg/dL (*n* = 3,033)	153.0 ± 38.4	150.4 ± 34.2	150.7 ± 37.4	158.0 ± 42.5	<0.0001
LDL cholesterol, mg/dL (*n* = 2,676)	86.0 ± 33.7	81.0 ± 30.2	86.0 ± 32.6	90.6 ± 37.1	<0.0001
LDL cholesterol ≤ 70 mg/dl, *n* (%)	901 (33.7)	316 (37.9)	310 (32.7)	275 (30.7)	0.005
LDL cholesterol ≤ 55 mg/dl, *n* (%)	395 (14.8)	141 (16.9)	128 (13.5)	126 (14.1)	0.10
LDL cholesterol ≤ 40 mg/dl, *n* (%)	107 (4.0)	33 (4.0)	40 (4.2)	34 (3.8)	0.89
HDL cholesterol, mg/dL	45.5 ± 13.6	56.6 ± 14.9	44.4 ± 8.9	36.3 ± 7.8	<0.0001
Triglycerides, mg/dL	112 (85-152)	76 (63-89)	110 (96-129)	170 (144-204)	<0.0001
Glycaemia, mg/dL (*n* = 2,742)	114.3 ± 36.0	109.3 ± 31.9	113.3 ± 35.4	120.3 ± 39.2	<0.0001
Uric acid, mg/dL (*n* = 1,968)	5.7 ± 1.7	5.4 ± 1.6	5.7 ± 1.6	6.1 ± 1.6	<0.0001

*BMI, body mass index; CABG, coronary artery by-pass grafting; COPD, chronic obstructive pulmonary disease; DBP, diastolic blood pressure; Hb, hemoglobin; HDL, high density lipoprotein; HR, heart rate; LDL, low density lipoprotein; LVEF, left ventricular ejection fraction; MI, myocardial infarction; PCI, percutaneous coronary intervention; SBP, systolic blood pressure; TIA, transient ischemic attack*.

* *Dialysis, history of renal transplant or creatinine levels >1.5 mg/dL*.

At the time of enrollment, a statin was prescribed in 2,907 (94.8%) patients: 921 (95.2%) in the low, 1,015 (94.8%) in the middle and 971 (94–5%) in the high tertile of TG/HDL-C ratio (*p* = 0.73). Atorvastatin was the most prescribed statin compound (69.1%), followed by simvastatin (12.1%) and rosuvastatin (11.6%), without significative differences between the tertiles of TG/HDL-C ratio. The median dosages of statins prescribed in the 3 groups of patients are shown in Supplementary Table 2.

Omega-3 fatty acids were prescribed in 12.5, 11.6, and 22.5% in the low, middle and high tertile of TG/HDL-C ratio, respectively (*p* < 0.0001).

**Figure 1:**
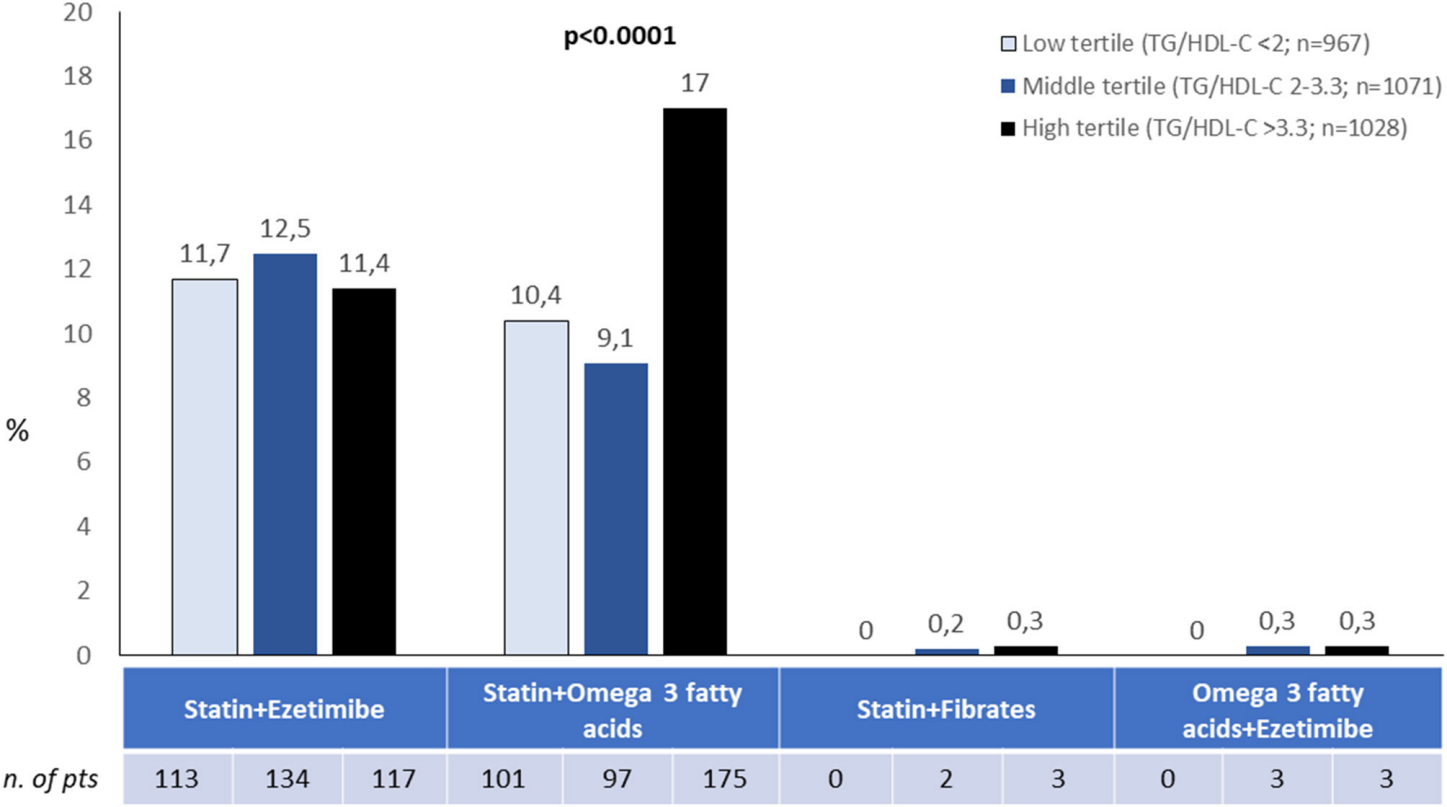
Associations of lipid lowering strategies according to tertiles of TG/HDL ratio. Other possible combinations not shown were used in <0.5% of cases.

The association of statin and ezetimibe was prescribed in 11.9% and statin and omega-3 fatty acids in 12.2% of patients, with a significantly higher prevalence for the latter among those in the higher tertile of TG/HDL-C ratio, while other associations of cholesterol lowering agents were not frequently employed (Figure 1).

**Figure 2:**
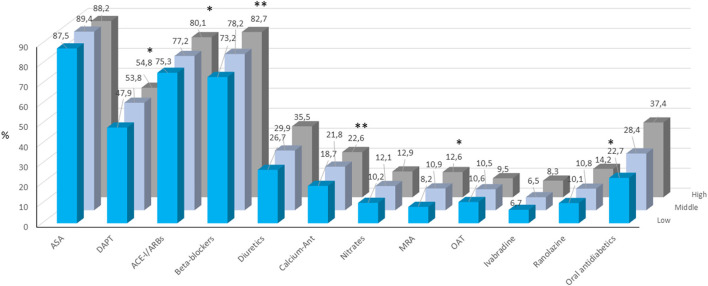
Pharmacological treatment at enrolment in patients included the three TG/HDL-C tertiles. ACE-I, angiotensin converting enzyme inhibitors; ARB, angiotensin receptor blockers; ASA, acetylsalicylic acid; CCA, calcium channels antagonists; MRA, mineralocorticoid receptor antagonist; OAT, oral anticoagulant therapy. *p < 0.05; **p < 0.0001.

The other pharmacological therapies prescribed in the three groups are shown in Figure 2. A dual antiplatelet therapy, ACE-inhibitors or angiotensin II receptor blockers, beta-blockers, calcium channel antagonists, mineralocorticoid receptor antagonists and ranolazine were more frequently prescribed in patients in middle and high compared to low TG/HDL ratio tertiles.

Notably, an OMT was prescribed in 65.9, 71.4, and 75.6% in the low, middle and high tertile of TG/HDL-C ratio, respectively (*p* < 0.0001).

### Clinical Events at 1 Year

Data on follow-up were available for 2,904 (95%) patients included in the analysis. The persistence to high dose statin at follow-up was 94.4, 94.2, and 95.6% in the low, middle and high tertile of TG/HDL-C ratio, respectively (*p* = 0.41).

**Figure 3:**
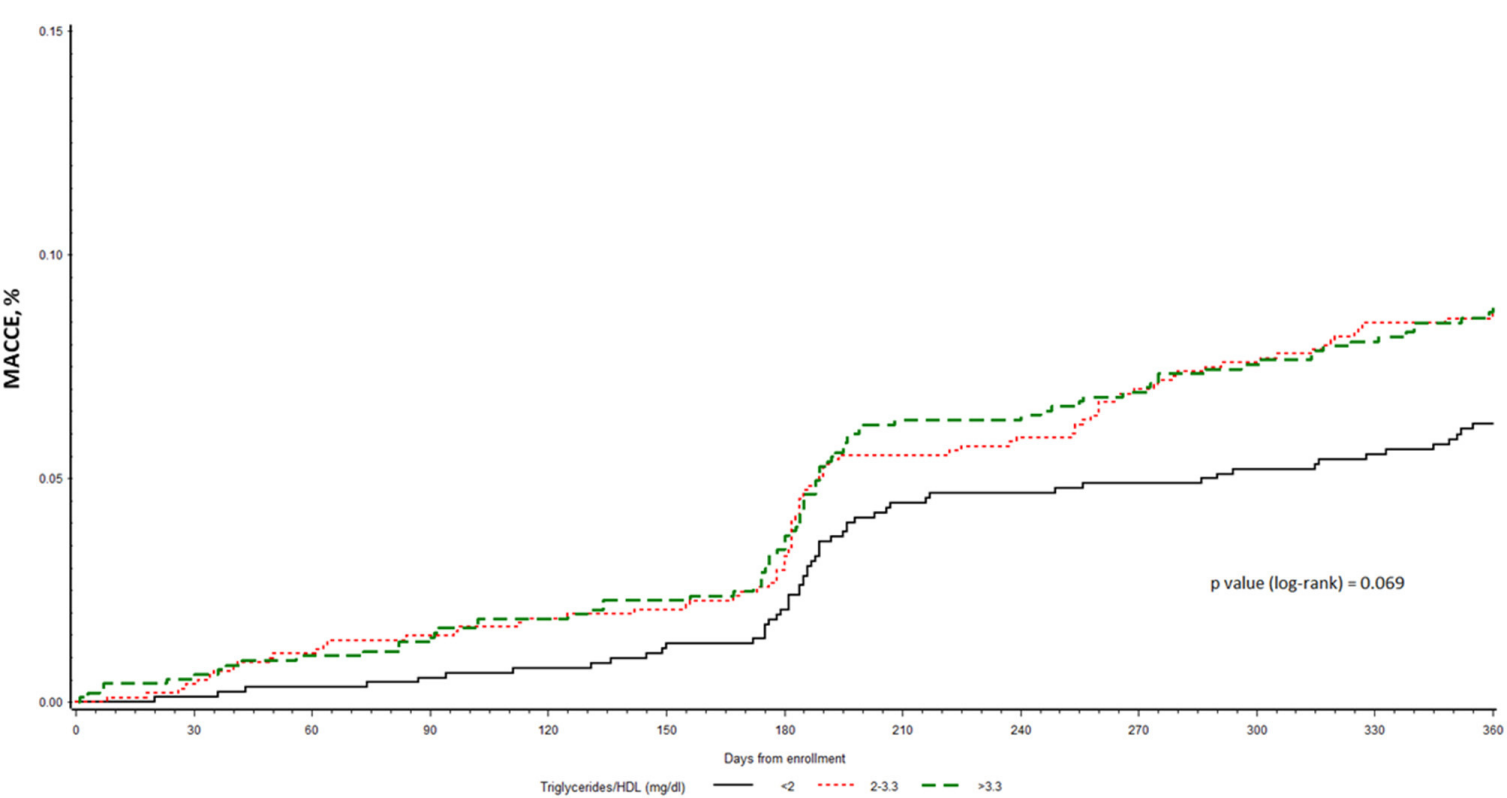
Kaplan-Meier curves for time to MACCE among the three subgroups of CCS patients at VHR stratified by TG/HDL-C tertiles.

At 1 year (median 369; IQR 362–378 days) from enrolment, MACCE was observed in 232 (7.9%) patients, and a higher incidence was observed in the higher tertile of TG/HDL ratio (6.0, 8.2, and 8.4% in the low, middle and high tertile, respectively), even though not statistically significant (*p* = 0.08). The single components of the MACCE respect to the 3 tertiles are reported in Table 2. The Kaplan-Meier curves of MACCE for the three tertiles are shown in Figure 3.

**Table 2:** Single components of MACCE at 1 year.

**Events, *n* (%)**	**Overall *n* = 3,066**	**Tertiles of TG/HDL-C ratio**			***p* value**
		**Low *n* = 967**	**Middle *n* = 1,071**	**High *n* = 1,028**	
All-cause death	47 (1.5)	10 (1.0)	19 (1.8)	18 (1.8)	0.31
Myocardial Infarction	24 (0.8)	6 (0.6)	9 (0.8)	9 (0.9)	0.78
Heart Failure	48 (1.6)	11 (1.1)	23 (2.2)	14 (1.4)	0.15
Stroke	13 (0.4)	5 (0.5)	2 (0.2)	6 (0.6)	0.33
Myocardial revascularization	135 (4.4)	34 (3.5)	48 (4.5)	53 (5.2)	0.20

At multivariable analysis, the TG/HDL-C ratio in tertiles did not result an independent predictor of the MACCE (*p* = 0.22) at 1-year follow-up (HR: 1.30; 95% CI: 0.93–1.82; *p* = 0.12 middle vs lower tertile, and HR: 1.22; 95% CI: 0.87–1.72; *p* = 0.25 higher vs lower), while other variables such as history of HF (HR: 2.11; 95% CI: 1.54–2.88; *p* < 0.0001), CKD (HR 2.17; 95% CI: 1.56–3.02; *p* < 0.0001) and LDL-C target achievement (HR: 0.75; 95% CI: 0.55–1.01; *p* = 0.04) resulted as independent predictors of the MACCE.

## Discussion

In a large cohort of CCS patients at VHR enrolled in the START registry in Italy, we found that patients in the highest tertile of TG/HDL-C ratio were predominantly males and significantly younger, and presented more often risk factors such as DM, smoking, hypertension, CKD and higher levels of glycaemia, serum uric acid, total and LDL cholesterol compared to patients in the lower tertiles, i.e., low and middle. At 1 year from enrolment, the incidence of MACCE did not statistically differ between the 3 groups though it was higher in the high tertile compared to the low and middle one. At multivariable analysis, the TG/HDL-C ratio in tertiles did not emerge an independent predictor of the MACCE at 1-year follow-up.

Several studies have reported that the combination of high TG and low HDL-C level was a predictor of cardiovascular disease independent of LDL-C level (1–4, 15). Moreover, combining the two lipid measures into one as the ratio of TG to HDL-C has been proved to be a reliable indicator for metabolic syndrome, insulin resistance, with direct implications in endothelial damage and atherosclerosis (5, 6, 16, 17). Reference values for TG/HDL-C ratio and its association with cardiometabolic diseases in a mixed adult population were also recently identified (18).

In addition, an elevated TG/HDL-C ratio, which has been proposed as an easily obtainable marker of atherogenic dyslipidemia, has been associated with adverse long-term cardiovascular outcomes and all-cause mortality in secondary prevention studies performed in gender-specific with suspected myocardial ischemia (4) or small country-specific (19) high-risk patients, as well as in patients presenting with ACS (9, 20). Poor data were available on the predictive role of TG/HDL-C ratio in patients with established CCS, particularly in those at VHR.

Recently, in a prospective, multicenter study enrolling 355 patients with stable angina at intermediate-low risk referred to coronary computed tomography angiography (CTA) and followed for 4.5 years, patients with a higher TG/HDL-C ratio presented a higher coronary atherosclerosis burden (21). In addition, both a higher TG/HDL-C ratio and the coronary CTA score were additional predictors of worse outcome, independently of other cardiovascular risk factors, the presence of obstructive CAD or ischemia (21). In contrast to these controlled studies, VHR CCS patients included in our registry were undertreated with statin therapies and were followed up for a short period of time. This may partly explain the lack of prognostic predictive value of the TG / HDL-C ratio observed in our analysis.

In fact, though the incidence of patients with MACCE was higher in the high tertile as compared to the low and middle one, a significative difference among the tertiles was not observed, and, at multivariable analysis, only history of HF and CKD resulted as independent predictors of the MACCE. However, it should be noted that a large majority of our study patients (71.1%) were receiving an OMT: 65.9%, 71.4 and 75.6% (*p* < 0.0001) in the low, middle and high tertile of TG/HDL-C ratio, respectively. Thus, the more widely represented OMT in the high tertile of TG/HDL-C ratio may partly account for the results. Moreover, the follow-up at 1 year was probably too short to observe a significant impact of TG/HDL-C ratio on clinical outcome. Indeed, even in a recent study in ACS patients with STEMI (9) a high TG/HDL-C ratio did not impact on 30-day and 1-year outcomes suggesting that a longer period is needed to reveal significant differences, as has been shown in other previous studies (15, 16, 19, 20).

### Study Limitations

Our study must be evaluated in the light of some limitations. First, data reported in the present analysis are limited to the time of enrolment and we do not have data on long-term persistence to prescribed therapies, their changes and relative outcomes. Nevertheless, a clinical follow-up at 1 year from enrolment in the START study showed a persistence to OMT therapy higher than 90% (11). In addition, we considered values of TG and HDL-C at enrolment. Therefore, changes in statins regimen and dosing could have occurred later influencing the subsequent TG/HDL-C ratios. Finally, even if the participating centers were asked to include in the registry all consecutive patients with SCC, we were not able to verify the enrolment process, due to the absence of administrative auditing.

## Conclusions

In the present large, nationwide cohort of consecutive CCS patients at VHR, a high TG/HD ratio did not emerge as independent predictor of cardio-cerebrovascular events at 1 year. Further larger studies with a longer follow-up period are needed to better define the clinical and prognostic role of TG/HDL ratio in CCS.

## Data Availability Statement

The raw data supporting the conclusions of this article will be made available by the authors, without undue reservation.

## Ethics Statement

The studies involving human participants were reviewed and approved by San Camillo-Forlanini, Rome. The patients/participants provided their written informed consent to participate in this study.

## Author Contributions

LD and PT drafted the manuscript. LG analyzed the data. All other authors critically revised the article. All authors contributed to the article and approved the submitted version.

## Funding

The sponsor of both studies was the Heart Care Foundation, a non-profit independent organization, which also owns the database. Database management, quality control of the data and data analyses were under the responsibility of the ANMCO Research Center Heart Care Foundation. The realization of this sub-analysis of the START study and the publication of its results was partially supported by an unrestricted grant by Menarini, Italy. No compensations were provided to participating sites, investigators, nor members of the Steering Committee. The Steering Committee had full access to all of the data and takes complete responsibility for the integrity of the data and the accuracy of the data analysis.

## Conflict of Interest

The authors declare that the research was conducted in the absence of any commercial or financial relationships that could be construed as a potential conflict of interest.

## Publisher's Note

All claims expressed in this article are solely those of the authors and do not necessarily represent those of their affiliated organizations, or those of the publisher, the editors and the reviewers. Any product that may be evaluated in this article, or claim that may be made by its manufacturer, is not guaranteed or endorsed by the publisher.
