# Supplementary material for: Clinical Impact and Prognostic Role of Triglyceride to High-Density Lipoprotein Cholesterol Ratio in Patients With Chronic Coronary Syndromes at Very High Risk: Insights From the START Study
Source: Front Cardiovasc Med. 2022 Apr 13;9:874087. doi: 10.3389/fcvm.2022.874087 (PMC9043517; doi:10.3389/fcvm.2022.874087)
Supplement: Supplementary file 1 [file Data_Sheet_1.pdf]

Suppl Table 1. Baseline characteristics of VHR patients (n=4751) with TG and HDL-C available vs not available

	TG and HDL-C available (n=3066)	TG and/or HDL-C not available (n=1685)	p
Age (years), mean ± SD	67.2±10.2	68.4±11.1	0.0003
Females, n (%)	555 (18.1)	359 (21.3)	0.007
BMI (kg/m^2), mean ± SD	27.4±4.0	27.3±4.1	0.35
**Risk factors and comorbidities, n (%)**			
Active smokers	572 (18.7)	249 (14.8)	0.0007
Diabetes mellitus	1025 (33.4)	531 (31.5)	0.18
Hypertension	2430 (79.3)	1359 (80.7)	0.25
Chronic renal dysfunction*	365 (11.9)	222 (13.2)	0.20
Peripheral artery disease	309 (10.1)	142 (8.4)	0.06
COPD	359 (11.7)	212 (12.6)	0.38
Malignancy	186 (6.1)	125 (7.4)	0.07
**Cardiovascular history, n (%)**			
Previous stroke/TIA	180 (5.9)	96 (5.7)	0.81
History of major bleeding	61 (2.0)	31 (1.8)	0.72
Atrial fibrillation	418 (13.6)	249 (14.8)	0.28
History of heart failure	415 (13.5)	246 (14.6)	0.31
Prior MI	2190 (71.4)	1234 (73.2)	0.18
Previous PCI/CABG	2582 (84.2)	1384 (82.1)	0.07

* Dialysis, history of renal transplant or creatinine levels >1.5 mg/dL.

Suppl Table 2. Median (IQR) dosages of statins prescribed at enrollment in VHR patients with TG and HDL-C available.

	Overall n=3066	Tertiles of TG/HDL-C ratio			p
		Low (<2) n=967	Middle (2-3.3) n=1071	High (>3.3) n=1028	
Atorvastatin	40 (20-40)	40 (20-40)	40 (20-40)	40 (40-40)	0.0003
Fluvastatin	80 (80-80)	45 (10-80)	80 (80-80)	80 (80-80)	0.47
Lovastatin	40 (20-40)	20 (20-40)	40 (40-40)	20 (20-40)	0.21
Pravastatin	40 (40-40)	40 (40-40)	40 (40-40)	40 (20-40)	0.73
Rosuvastatin	10 (10-20)	10 (10-20)	10 (10-20)	10 (10-20)	0.82
Simvastatin	20 (20-40)	20 (20-40)	20 (20-40)	20 (20-40)	0.36

Appendix

Steering Committee

L De Luca (Chairman), MM Gulizia (co-chairman), PL Temporelli, C Riccio, F Colivicchi, AF Amico, D Formigli, G Geraci, A Di Lenarda

Executive Committee

L De Luca, AP Maggioni, D Lucci

Coordinating Center

ANMCO Research Center (AP Maggioni, D Lucci, A Lorimer, G Orsini, L Gonzini, G Fabbri, P Priami)

Participating Centers and Investigators

Trieste, Maggiore (P Maras, F Ramani); Pavia, Istituto di Cura Città di Pavia (C Falcone, I Passarelli, S Mauri); Napoli, AORN Colli-Monaldi, UOC Cardiologia-SUN (P Calabrò, R Bianchi, G Di Palma); Caserta, AO S. Anna e S. Sebastiano, UO Cardiologia-UTIC (F Mascia, A Vetrano, A Fusco); Piedimonte Matese (E Proia); Roma, San Filippo Neri (F Colivicchi, A Aiello); Roma, European Hospital (F Tomai, R Licitra, A Petrolini); Santa Maria Capua Vetere (B Bosco); Lecce, V. Fazzi, UO Cardiologia (F Magliari, M Callerame, T Mazzella); Vittoria (GV Lettica, G Coco, F Incao); Città di Castello (L Marinacci, S D'Addario); Sanremo (SN Tartaglione, S Ubaldi, FA Sanchez); Avola (P Costa, G Manca, M Failla); Benevento, AO G. Rummo (M Scherillo, V Procaccini, D Formigli); Bergamo, ASST Papa Giovanni XXIII (M Senni, EM Luminita); Cagliari, SS Trinità (P Bonomo, C Mossa, S Corda); Campobasso, Cardarelli (AR Colavita, G Trevisonno, G Vizzari); Cariati (N Cosentino, C Formaro); Corato (C Paolillo, IL Nalin); Cosenza, Annunziata (FM De Rosa, F Fontana, GF Fuscaldo); Cremona (E Passamonti, E Bertella, EV Calvaruso); Faenza (E Varani, F Tani, G Cicchitelli); Fermo (D Gabrielli, P Paoloni, A Marziali); Ferrara (G Campo, M Tebaldi, S Biscaglia); Foggia, Riuniti (M Di Biase, ND Brunetti, AM Gallotta); Gorizia (L Mattei, R Marini, F Balsemin); Magenta (M D'Urbano, R Naio, P Vicinelli); Massa, Apuane (G Arena, M Mazzini, N Gigli); Melito di Porto Salvo (B Miserrafiti, A Monopoli); Monza, Policlinico (A Mortara, P Delfino, MM Chioffi); Novara, AOU Maggiore della Carità, SCDU Clinica Cardiologica-Cardiologia I (P Marino, M Gravellone, L Barbieri); Palermo, AOR Villa Sofia-Cervello (A Ledda, G Geraci, MG Carmina); Pavia, IRCCS Policlinico San Matteo (AE Raisaro, C Di Giacomo, A Somaschini); Potenza, San Carlo, SSD Card. Riab. (ML Fasano, M Sannazzaro, R Arcieri); Reggio Emilia, S.M. Nuova (M Pantaleoni, C Leuzzi, G Gorlato); Roma, Santo Spirito (G Greco, A Chiera); Rozzano (TA Ammaturo, G Malanchini, MP Del Corral); Battipaglia (L Tedesco); Lecce, Casa di Cura Petrucciani (S Pede, LG Urso); Salerno (F Piscione, G Galasso); Varese, Circolo e Fond. Macchi (S Provasoli); Aversa (L Fattore, G Lucca); Grosseto (A Cresti); Caserta, AO S. Anna e S. Sebastiano, Cardiologia e Riabil. Cardiol. (A Cardillo); Pomezia (MS Fera, F Vennettilli); Roma, Umberto Primo, Cardiologia B - Cardiologia e Angiologia (C Gaudio, V Paravati); Bari, San Paolo (P Caldarola, N Locuratolo); Camposampiero (R Verlato, F De Conti); Conegliano (G Turiano, G Preti); Ascoli Piceno (L Moretti, S Silenzi); Lecce, V. Fazzi, UO Card. Interventistica-Emod. (G Colonna, A Picciolo); Ragusa (A Nicosia, C Cascone); Roma, Campus Biomedico (G Di Sciascio, F Mangiacapra); San Giovanni Rotondo (A Russo, S Mastroianno); Carate Brianza (G Esposito); Cortona (F Cosmi, S D'Orazio); Jesi (C Costantini, A Lanari); Giugliano In Campania (P De Rosa, L Esposito); Arzignano (C Bilato, C Dalla Valle); Pavia, ICS Maugeri (M Ceresa, E Colombo); Reggio Calabria, Bianchi Melacrino Morelli (V Pennisi, G Casciola); Udine, Santa Maria Misericordia (M Driussi, T Bisceglia); Lumezzane (S Scalvini, F Rivadossi); Roma, Sant'Andrea (M Volpe, F Comito); Tradate, Galmarini (D Scorzoni, P Grimoldi); Cassano delle Murge (R Lagioia, D Santoro); Osio Sotto (N De Cesare, T Comotti); Legnano (A Poli, P Martina); Locri (MF Musolino, EI Multari); Feltre (G Bilardo, G Scalchi); Isernia (C Olivieri, F Caranci); San Vito al Tagliamento (D Pavan, G Ganci); Senigallia (A Mariani, E Falchetti); Avellino (T Lanzillo, A Caccavale); Novara, AOU Maggiore della Carità, Cardiologia II (AS Bongo, A Rizzi); Siena (R Favilli, S Maffei); Napoli, San Gennaro (M Mallardo, C Fulgione); Thiene (F Bordin); Trento, Santa Chiara (R Bonmassari, E Battaia); Troina (A Puzzo); Chioggia (G Vianello); Poggibonsi (A D'Arpino, M Romei); Albano Laziale, Albano-Genzano (G Pajes, S Petronzelli); Cesena (F Ghezzi); Monfalcone (S Brigido, L Pignatelli); Torino, Maria Pia Hospital (E Brscic, P Sori); Barletta (M Russo, E Biancolillo); Brindisi (G Ignone, NA De Giorgio); Formia (C Campaniello, P Ponticelli); Milano, San Raffaele (A Margonato, S Gerosa); Agrigento (A Cutaia, C Casalicchio); Andria (F Bartolomucci, C Larosa); Molfetta (T Spadafina, A Putignano); Orvieto (R De Cristofaro, L Bernardi); Viterbo (L Sommariva, A Celestini); Alessandria, Clinica Città di Alessandria (CM Bertucci, M Marchetti); Belluno (E Franceschini Grisolia, C Ammendolea); Casalmaggiore (M Carini); Fabriano (P Scipione, M Politano); Marsala (G Rubino, C Reina); Mormanno (N Peccerillo); Pescara (L Paloscia, A D'Alleva); Sarzana (R Petacchi); Aprilia (M Pignalosa, D Lucchetti); Boscotrecase (F Di Palma, RA La Mastra); Galatina (AF Amico, M De Filippis); Gavardo (B

Fontanella, G Zanini); Lido di Camaiore (G Casolo, J Del Meglio); San Benedetto del Tronto, Madonna del Soccorso (VM Parato, E Genovesi); Somma Lombardo (A D'Alimonte, A Miglioranza); Latina, Polo Ospedaliero Integrato (N Alessandri, F Moscariello); Napoli, AORN Cardarelli (C Mauro, A Sasso); Napoli, AORN Colli-Monaldi, UOC Cardiologia (P Caso, C Petrillo); Teramo (C Napoletano, SR Paparoni); Rieti (V Bernardo, R Serdoz); Roccadaspide (R Rotunno, I Oppo); Taranto, Casa di Cura Villa Verde (A Aloisio, A Aurelio); Augusta (G Licciardello, L Cassaniti); Catania, Garibaldi-Nesima (MM Gulizia, GM Francese); Veruno (C Marcassa, PL Temporelli); Vigevano, Civile (R Villani, F Zorzoli); Polistena (F Mileto, M De Vecchis); Copertino (AF Amico, D Scolozzi); Genova, Padre Antero Micone (G Lupi, D Caruso); Palermo, Casa di Cura Candela (E Rebulla, B La Fata); San Bonifacio (M Anselmi, P Girardi); Alcamo (E Borruso, G Ferrantelli); Cento (B Sassone, S Bressan); Ciriè (M Capriolo, E Pelissero); Lugo (M Piancastelli, M Gobbi); Manduria (F Cocco, MG Bruno); Massa, FTGM - Stabilimento di Massa (S Berti, G Lo Surdo); Roma, San Camillo, Cardiologia 2 - Ex Cardio 3 (P Tanzi, R De Rosa); Scorrano (E Vilei, MR De Iaco); Venezia (G Grassi, C Zanella); Castel Volturno (L Marullo, G Alfano); Lamezia Terme (P Pelaggi, R Talarico); Napoli, Loreto Mare (B Tuccillo, L Irace); Roma, Aurelia Hospital (F Proietti, G Di Croce); Sessa Aurunca (L Di Lorenzo, A Zarrilli); Imperia (M Bongini, A Ranise); Ivrea (A Aprile, C Fornengo); Melfi (V Capogrosso, A Tranghese); Napoli, Clinica Mediterranea (B Golia, A Marziano); Rovigo (L Roncon, C Picariello); Sassuolo (E Bagni, E Leci); Vallo della Lucania (G Gregorio, F Gatto); Frattamaggiore (F Piemonte, F Gervasio); Guastalla (A Navazio, E Guerri); Roma, Madre Giuseppina Vannini (E Belmonte, F Marino); Anzio (N Di Belardino, MR Di Nuzzo); Bari, Policlinico (M Epifani); Milano, San Carlo Borromeo (G Comolatti, B Conconi); Novara, Clinica San Gaudenzio (D Benea); Nuoro (G Casu, P Merella); San Giuseppe Vesuviano (MA Ammirati, VM Corrado); Civitanova Marche (D Spagnolo); Gallarate (SI Caico); Milano, Istituto Clinico Città Studi (S Bonizzato); Ravenna (M Margheri); Vercelli (L Corrado); Ancona, INRCA (R Antonicelli); Gela (C Ferrigno); Sant'Agata di Militello (A Merlino); Saronno (D Nassiacos); Sesto San Giovanni, IRCCS Policlinico Multimedica (A Antonelli); Siracusa, Umberto I, UOC Cardiologia e UTIC (A Marchese); Roma, San Camillo, UOC Cardiologia 1 (M Uguccioni); Cerignola (A Villella); Correggio (A Navazio); Piombino (S Bechi); Roma, Sandro Pertini (F Lo Bianco); San Donato Milanese, IRCCS Policlinico San Donato, UO Cardiologia con UTIC (F Bedogni); Tricase (L Negro); Vizzolo Predabissi (L Donato); Francavilla Fontana (D Statile); Pordenone, Ospedale di Pordenone, SOC Cardiologia (M Cassin); Roma, Umberto Primo, Malattie Cardiovascolari A (F Fedele); Tivoli (A Granatelli); Civitavecchia (S Calcagno); Gravedona (A Politi); Roma, San Pietro FBF (R Serdoz); Cagliari, AO Brotzu, SC Cardiologia (A Pani).
